# Pig Xenotransplantation in Beta Cell Replacement: Addressing Challenges and Harnessing Potential for Type 1 Diabetes Therapy

**DOI:** 10.3389/ti.2024.13122

**Published:** 2024-10-24

**Authors:** Lorenzo Piemonti, Antonio Citro, Valentina Tomajer, Stefano Partelli, Rossana Caldara

**Affiliations:** ^1^ Clinic Unit of Regenerative Medicine and Organ Transplants and Diabetes Research Institute, Istituto di Ricovero e Cura a Carattere Scientifico (IRCCS) Ospedale San Raffaele, Milan, Italy; ^2^ Diabetes Research Institute, Università Vita-Salute San Raffaele, Milan, Italy; ^3^ Pancreatic Surgery, Pancreas Translational and Clinical Research Center, Istituto di Ricovero e Cura a Carattere Scientifico (IRCCS) Ospedale San Raffaele, Milan, Italy

**Keywords:** porcine islets, xenotransplantation, type 1 diabetes, immunogenicity, regulatory framework

## Abstract

This opinion paper evaluates the potential of porcine islets as a promising alternative in beta cell replacement therapy for Type 1 Diabetes (T1D), juxtaposed with the current limitations of human donor islets. It analyzes the compatibility of pig islets with human glucose metabolism, their prospects as a limitless and high-quality source of beta cells, and the unique immunogenic challenges they present in xenotransplantation. Additionally, the paper discusses the regulatory and ethical considerations pertinent to the use of porcine islets. By synthesizing current research and expert perspectives, the paper highlights both the opportunities and significant barriers that need addressing to advance pig islets as a viable therapeutic option. The findings advocate for a balanced and forward-looking approach to the integration of pig islets in T1D treatment, underscoring the need for continued research and dialogue in this evolving field.

## Introduction

The path to curing Type 1 Diabetes (T1D) through beta cell replacement is filled with both promise and complexity [1, 2]. At its core, the strategy is simple and compelling: by restoring the insulin-producing function of pancreatic beta cells, one can tackle T1D at its root [3]. The use of islets from organ donors in these therapies has convincingly shown that such an approach can significantly improve or even temporarily reverse diabetes [4–8]. Yet, this achievement also highlights the critical hurdles that need to be cleared to reach a universal cure. The main challenges in beta cell replacement therapies involve finding a limitless supply of beta cells, reducing or avoiding the need for immunosuppressive drugs, and ensuring the transplanted cells survive and function over the long term [9]. The reliance on donor islets is severely limited by donor scarcity, variable islet quality, and the complexities tied to working with primary culture cells, which together pose a significant challenge to the broad applicability of beta cell replacement methods. The pursuit of alternative sources of cells holds potential; however, these alternatives should deliver distinct advantages over current options in terms of availability, quality consistency, and ease of handling to truly transform the landscape of beta cell replacement therapies. Given these foundational challenges, it becomes crucial to consider if pig islets could potentially offer advantages over traditional organ donor sources or alternative strategies, such as those involving the differentiation of pluripotent stem cells [10]. Addressing this issue effectively requires answering some key questions.

## How Compatible is the Glucose Metabolism Regulation of Pig Islets With Human Physiology for Transplantation Purposes?

The critical prerequisite for evaluating pig islets for transplantation is that the regulation of glucose metabolism by the pig islet must be compatible with human physiology [11]. While pig and human insulin are remarkably similar, differing only in the 30th amino acid of the β-chain, and despite the historical use of porcine insulin in treating human diabetes, there are differences in how porcine islets respond to glucose and other stimuli compared to human islets (summarize in Table 1) [12–22, 24–26]. Although the precise long-term consequences of these differences in pig islet function remain uncertain, they seem to align with the requirements for a short to medium-term approach.

**TABLE 1:** Comparative analysis of porcine and human islet function and potential in xenotransplantation.

Aspect	Details	Consequences
Insulin Response [12–16]	Porcine islets secrete 3–6 times less insulin than human islets in response to glucose	May require transplantation of more islets or genetic modification to meet human insulin requirements
Metabolic Control [17, 18]	Pigs have more glucose tolerance and lower basal insulin levels compared to humans	Adjustments in insulin therapy might be necessary post-transplantation to ensure metabolic control
Glucagon Response [19]	Porcine islets show a strong glucagon response to hypoglycemia, beneficial for clinical applications	Could enhance safety by preventing hypoglycemia in recipients
Diabetes Resistance [20–23]	Pigs are resistant to amyloidosis, unlike humans, possibly due to differences in IAPP sequence	Might reduce risk of islet dysfunction and extend longevity of the xenotransplant
Genetic Engineering [18, 24–26]	Human transgenes in pigs do not adversely affect glucose metabolism	Genetic modifications can make xenotransplantation a viable solution without disrupting glucose levels

## Is the Supply of Insulin-Producing Cells From Pig Islets a Limitless and Consistently High-Quality Source?

Bypassing the obstacles related to genetic adjustments and the circumvention of immune system rejection, the notion of pig islets serving as an endless and consistently superior source of insulin-producing cells encounters multiple critical areas in need of continuous improvement [27]. It is imperative to ensure the uniform quality and longevity of islet cells across various preparations, which mandates the formulation of uniform protocols for the extraction, refinement, and preservation of pig islets to maintain their operational effectiveness and durability post-transplant [28–30]. Additionally, the ability to upscale the production of pig islets poses an essential challenge [31, 32]. This encompasses the initiation of responsible and ethical practices in pig rearing, the advancement of proficient methods for islet extraction, and the creation of effective logistical solutions for their distribution [33]. These factors need to be refined to address the worldwide demand for insulin-producing cells while safeguarding animal wellbeing and ecological integrity. The ongoing debate (summarize in Table 2), indicating that after extensive research there still has not been a consensus on the most suitable pig age and strain for providing sufficiently viable isolated islet cells for clinical xenotransplantation [47–50], underscores the current limitations in achieving a limitless and consistent supply [51].

**TABLE 2:** Advantages and disadvantages of fetal, neonatal and adult pig islets for clinical xenotransplantation.

Aspect	Fetal pig islet-like cell clusters (ICCs)	Neonatal pig islets (NPIs)	Adult pig islets (APIs)
Source Age	Fetal	<14 days old	>12 weeks old
Isolation and Preparation Challenges [32, 34–37]	Straightforward; includes enzymatic digestion and culture	Comparable to ICCs, with effective recovery thanks to damage resistance	Echoes human islet isolation, accounting for donor pancreas condition, blood removal, and warm ischemia. Density gradient centrifugation with Ficoll and Iodixanol enhances yield and viability
Islet yield/pancreas (Beta cells %) [32, 34, 38]	7,000–10,000 (<10%)	25,000–50,000 (25%)	200,000–500,000 (>70%)
Maturation for *In Vivo* Functionality [39–45]	2–3 months maturity required for *in vivo* functionality	More responsive to glucose, leading to restoration of normoglycemia due to β cell expansion and differentiation	Immediate functionality post-transplantation
Safety	*In vivo* proliferation, minimal tumorigenic risk, low pathogen transmission risk	*In vivo* proliferation, minimal tumorigenic risk, low pathogen transmission risk	No *in vivo* proliferation or tumorigenic risk, low pathogen transmission risk
Cost and Practicality Considerations [31]	Not directly stated	Low cost of maintenance pre-pancreatectomy, simpler isolation, lower costs than APIs, but requires more donors for sufficient islets	High maintenance cost, isolation difficulty and cost, but greater yield of high-quality islets from retired breeders
Pig islet donor for a primate recipient	Not currently being considered for xenotransplantation (because of limited β-cell yield, poor insulin response to glucose and high destruction rate post-transplantation)	Requires a minimum of four neonatal donors per diabetic primate	A single adult donor may be sufficient
Pig islet donor for a human recipient		14–28 donors needed	1–2 donors sufficient
Clinical Application Potential [39, 46]		Limited by the need for multiple donors and additional maturation steps	Significant, with the potential for single-donor clinical transplantation

## Do Pig Islet Cells Possess Inherent Advantages in Terms of Immunogenicity for Xenotransplantation Purposes?

In principle, xenotransplantation of pig islets could present a less specific target for the recipient’s autoimmune response compared to human islets, due to the differences in cellular antigens between species. Thus, while the pig islet cells could still be recognized as foreign by the recipient’s immune system, they might not be specifically targeted by the autoreactive T cells that are involved in the autoimmune attack on native pancreatic beta cells. However, this potential advantage is complicated by the broad immune response against xenogeneic tissue, which includes not only adaptive immune responses [52–56] but also innate responses [57] and issues like the instant blood-mediated inflammatory reaction (IBMIR) [58–63], hyperacute rejection, and acute cellular rejection. These xenogeneic reactions can be strong and present significant barriers to the long-term survival and function of the transplanted islets. Furthermore, cross-reactivity between swine leukocyte antigen (SLA) and human anti-HLA-specific antibodies is another factor that complicates xenotransplantation [64]. It is well-known that human anti-HLA antibodies, especially in sensitized individuals, can sometimes bind to SLA molecules due to structural similarities [65, 66]. This cross-reactivity can lead to both innate and adaptive immune responses, potentially causing early rejection of the pig islets. While the basic concept of cross-reactivity is established and supported by various experimental studies [67–70], the extent to which cross-reactivity impacts clinical outcomes is still debated [71, 72]. The predictability of which specific human antibodies will cross-react with SLA, and how this cross-reactivity might vary among different individuals or pig donors, remains an area of ongoing research [73, 74]. There is also debate about the best strategies to mitigate these risks, including whether genetic modifications to reduce SLA expression or to incorporate human-like antigens in pigs are sufficient to prevent cross-reactivity [75]. Thus, while pig islet cells might have certain inherent advantages in terms of reduced targeting by autoreactive T cells, the risk of cross-reactivity with pre-existing anti-HLA antibodies adds a significant layer of complexity to the immunogenicity of xenotransplanted pig islets.

## Are Methods to Alleviate Xenogeneic Rejection More Feasible or Practical Compared to Those Used for Other Transplant Sources?

Alleviating xenogeneic rejection involves several unique and sophisticated approaches due to the significant biological differences between species. These methods can be categorized into three main strategies: gene editing of the donor (e.g., pigs), pharmacological immunosuppression, and physical barriers such as encapsulation. Focusing specifically on gene modification efforts, pig islet xenotransplantation indeed represents the most extensive application of genetic engineering in the realm of islet transplantation (Table 3) [91]. The most extensively modified animals have undergone 69 genomic alterations, which include the removal of glycan antigens, the enhancement of human transgene expression, and the deactivation of porcine endogenous retroviruses [92]. This extensive level of genetic editing is primarily possible because of the wider array of modifications that are both ethically and technically feasible in pigs, in contrast to what is possible with human islets or stem cells. Conversely, evidence suggests that traditional immunosuppressive treatments, such as those based on tacrolimus, are less effective at managing the adaptive immune response against pig xenografts [93]. Instead, targeting the CD40/CD154 T-cell co-stimulation pathway has shown greater efficacy [94]. Notably, islets from adult wild-type (non-genetically modified) pigs have successfully functioned in diabetic non-human primates (NHPs) treated with anti-CD154 monoclonal antibody-based immunosuppression for up to 965 days [95]. Nonetheless, genetically editing pigs could potentially achieve comparable or superior outcomes with less aggressive immunosuppression (Table 4). The concern, however, lies in the intensity of immunosuppressive protocols required, particularly for conditions requiring long-term management like diabetes. Islet encapsulation may offer greater immediate benefits for pig islet transplantation compared to human islets [110]. This is due to pig islets’ higher immunogenicity and the ample supply they offer, which is critical for overcoming xenotransplantation’s unique challenges [111]. While both pig and human islet transplants can benefit from encapsulation technologies, the necessity and feasibility of these strategies might be more pronounced for pig islets to ensure successful transplantation outcomes. Although significant progress has been made [112–114], most strategies are yet to meet the criteria of obtaining sustainable and consistent diabetes management for >6 months in preclinical trials before they can be introduced in human clinical trials.

**TABLE 3:** Targeted deletions/insertions in pig genome for xenotransplantation.

Gene modification	Purpose	Impact on transplantation
α1,3-Galactosyltransferase Knockout (GTKO) [69, 76]	Eliminates α-Gal epitopes to reduce hyperacute and acute vascular rejection	Significantly decreases antibody-mediated rejection; first major step towards viable xenotransplants. Knockout of the genes for the 3 glycan xenoantigens (providing triple-knockout, [TKO] pigs) is generally considered the basis of the pigs that will be sources of organs and cells for clinical transplantation
Cytidine monophosphate-N-acetylneuraminic acid hydroxylase Knockout (CMAH-KO) [77]	Eliminates Neu5Gc to reduce hyperacute and acute vascular rejection	
β-1,4N-acetylgalactosaminyltransferase Knockout (β4GalNT2-KO) [69]	Eliminates Sd(a) to reduce hyperacute and acute vascular rejection	
CD55 (DAF) Transgenic [78]	Regulates complement activation, reducing complement-mediated cell lysis	Enhances graft survival by protecting against complement-mediated damage
CD59 Transgenic [79]	Prevents the formation of the Membrane Attack Complex (MAC), protecting cells from complement-mediated lysis	Further protects xenografts from complement-mediated injury, complementing CD55 effects
CD46 (MCP) Transgenic [80]	Regulates complement activation on cell surfaces	Provides broad protection against complement activation, enhancing graft protection
Human Heme Oxygenase-1 (HO-1) Transgenic [81]	Provides cytoprotective, anti-inflammatory, and anti-apoptotic effects	Reduces ischemia/reperfusion injury and improves graft survival by mitigating acute inflammatory responses
Human Thrombomodulin (hTBM) Transgenic [82]	Modifies the coagulation cascade to reduce thrombosis in the graft	Addresses the issue of coagulation dysregulation in xenotransplants, improving graft function and longevity
α1,2-Fucosyltransferase (H Transferase) Transgenic [83]	Masks non-Gal antigens to further reduce antibody-mediated rejection	Complements GTKO by masking remaining xenoantigens, further reducing the immune response against the xenograft
SLA Class I and II Knockout [84, 85]	Reduces the expression of swine leukocyte antigens, decreasing T-cell mediated rejection	Aims to minimize direct T-cell recognition and response, lowering the risk of cellular rejection and the cross-reactivity of anti-HLA antibodies with SLA antigens
CD39 Transgenic [86]	Increases ATP and ADP hydrolysis, reducing platelet aggregation and thrombosis	Targets the prevention of thrombotic microangiopathy, promoting longer graft survival
PD-L1 Transgenic [87]	Inhibits T-cell activation and proliferation by engaging PD-1 on T cells	Contributes to creating an immunotolerant environment around the xenograft, reducing cellular rejection
HLA-E and HLA-G Transgenic [88, 89]	Engages inhibitory receptors on NK cells and certain T cells, reducing their activity	Aims to protect xenografts from NK cell-mediated damage and modulate T-cell responses, enhancing tolerance
CTLA4-Ig Transgenic [90]	Provides local immunosuppression by blocking costimulatory signals necessary for T-cell activation	Reduces the need for systemic immunosuppression, lowering side effects while protecting the graft

**TABLE 4:** Immunosuppressive protocols associated with prolonged periods of insulin-independence and islet xenograft survival.

	Donor	Recipient	Islets (IEQ/kg)	Immunosuppression	Max graft survival (days)	Ref.
Wild type	Adult SNU Miniature Pig	STZ-induced diabetic rhesus monkeys	96,090	Anti-CD40 mAbs (2C10R4), Sirolimus, ATG, CVF, Tacrolimus, Adalimumab, Methylprednisolone	>320	[96]
			93,575	ATG, sirolimus, tacrolimus, anti-CD40 mAb, tocilizumab, CVF, adalimumab	176	[97]
			100,000	ATG, anti-CD154 mAbs (5C8), Sirolimus, CVF, TNF-a-neutralizing mAb (adalimumab)	603	[95]
			50,000–150,000	ATG, Rituximab, Belimumab, Sirolimus, Tacrolimus, Tofacitinib, Adalimumab, Anakinra, CVF, IVIg	201	[98]
			100,000	ATG, Belimumab, Sirolimus, Tacrolimus, Abatacept, Tofacitinib, Adalimumab, Anakinra, Tocilizumab, IVIg, Aspirin	222	[99]
			100,000	ATG, CVF, anti-CD154 mAbs (5C8), Anti-CD40 mAbs (2C10R4), Sirolimus, TNF-a-neutralizing mAb (adalimumab), Treg	965	[100]
			93,575	ATG, sirolimus, tacrolimus, anti-CD40 mAb, tocilizumab (IL-6 receptor antagonist), CVF, adalimumab	176	[97]
	Neonatal Duroc or Large White Crossbreeds	Rhesus macaques s/p pancreatectomy	50,000	Anti-CD 154 mAb, basiliximab, sirolimus, belatacept	>260	[101]
			50,000	Anti-CD40 mAbs (Chi220), aIL-2R (Basiliximab), Belatacept, Sirolimus	>203	[102]
		Rhesus macaques/STZ	50,000	MMF + CTLA4-Ig + LFA-3-Ig + anti-IL-2R + anti-LFA-1	114	[103]
	Adult Outbred swine and inbred miniature swine	Cynomolgus monkeys/STZ	25,000	Basilixumab, FTY720 or tacrolimus, everolimus, anti-CD154 mAb, leflunomide	>187	[104]
		Rhesus macaques/STZ	up to 280,000	Anti-ICAM-1 mAbs (MD-3), anti-CD154 mAbs (5C8), Sirolimus, TNF-a-neutralizing mAb (adalimumab), Anakinra, Ganciclovir, Clopidogrel, Heparin	520	[105]
Genetic Modifications	Adult GTKO hCD46 hCD39 or similar	Cynomolgus monkeys/STZ	85,000	ATG, Anti-CD154 mAbs (h5c8), MMF, Dextran sulfate, Prostacyclin, Methylprednisolone, Aspirin, Ganciclovir, Famotidine, Heparin	365	[22]
	Neonatal GTKO hCD55 hCD59 HT	Nondiabetic baboons	17,889	ATG, MMF, tacrolimus	30	[106]
	Adult GTKO	Cynomolgus monkeys/STZ	40,000	ATG, tacrolimus, rapamycin, anti-CD154 mAb, MMF	>58	[107]
	Adult, hCD46	Cynomolgus monkeys/STZ	85–100,000	ATG, Anti-CD154 mAb (ABI7953), Dextran sulfate, Methylprednisolone, Aspirin, Prostacyclin	396	[108]
	Fetal, hCD55	Cynomolgus monkeys	Not reported	Cyclosporine + steroids + cyclophosphamide or brequinar	7–40	[109]

## Is There Evidence That Pig Islets Are More Effective in Regulating Human Blood Sugar Levels Compared to Other Sources in Clinical Trials?

Beyond the pioneering studies by Groth et al. [115] and Wang et al. [116], which examined free islet xenotransplantation, there’s a notable scarcity of clinical trials in this area (Table 5). Instead, much of the focus has been on encapsulated islets transplanted without the need for immunosuppression [117]. These endeavors have yet to achieve unequivocal success. In several instances, improvements in blood sugar management could be attributed to rigorous medical oversight, including dietary changes, strict glucose monitoring, and specialized healthcare, rather than the transplanted pig islets themselves. However, research led by Matsumoto et al. stands out, having successfully reduced HbA1c levels significantly over 600 days in recipients of encapsulated pig islets, without resorting to immunosuppressive medications [118, 119]. Despite minimal side effects reported, the quest for more dependable and enhanced results continues. When these findings are weighed against the outcomes from human islet and stem cell-derived islet transplants, it’s clear that transplants using human islets are currently the most effective for clinical management of diabetes, primarily due to their significant impact on glycemic control [6]. While pig islet xenotransplantation holds potential and has shown varied degrees of success, it still needs further development for it to be consistently reliable and widely applicable. On the other hand, islets derived from stem cells hold immense promise due to their potentially unlimited availability and fewer compatibility challenges. However, while their effectiveness has been shown in limited trials [120], confirmation of their clinical efficacy is still awaiting results from larger-scale studies.

**TABLE 5:** Clinical trials involving porcine islets.

Trial	Islet source	Recipient details	Islet type	Average number of islets (IEQ/kg)	Site of transplant	Immunosuppression	Outcome
Groth et al. [115]	Swedish Landrace	T1D with kidney transplant (n = 10)	Fetal Free	200,000–1 million	Kidney capsule or intraportal	ATG, 15-deoxyspergualin, cyclosporine, prednisolone, azathioprine	No improvement in glycemic control
Elliott et al. [124, 137]	Cross-White Breed	T1D with and without kidney transplant (n = 1 each)	Neonatal Encapsulated	15,000	Peritoneum	None (n = 1) and Standard (n = 1)	Short-term insulin requirement and HbA1c improvement, no PERV transmission
Valdes-Gonzalez et al. [138, 139]	New Zealand bred	T1D adolescents (n = 12)	Neonatal with Sertoli cells	13,000–20,000 (first transplant)	Subcutaneous	None	Half achieved insulin reduction, improvement in HbA1c and less chronic complications
Wang et al. [116]	Xeno-1	T1D (n = 21)	Neonatal Free	55,000	Intraportal	Comprehensive regimen	Reduction in insulin requirements, improvement in HbA1c, no PERV transmission
Matsumoto et al. [118]	Auckland Island	T1D (n = 14)	Neonatal Encapsulated	5,000–20,000	Peritoneum	None	Reduction in unaware hypoglycemic events, minimal HbA1c or insulin dose change
Matsumoto et al. [119]	Auckland Island	T1D (n = 8)	Neonatal Encapsulated	5,000 and 10,000	Peritoneum	None	

## Is There Evidence to Suggest that Porcine Islets Are Safer and More Regulatorily Straightforward Compared to Alternative Sources?

The safety and regulatory ease of using porcine islets compared to other sources such as human islets or stem cell-derived islets are areas of active research and debate. Adult porcine pancreatic islets could represent a safer and more effective alternative for therapeutic use than stem cells, due to their complete and immediate hormonal compatibility with human physiology and lower oncogenic risk, as they skip the need for differentiation and do not proliferate. While neonatal and fetal pig islets are less ideal due to their immature and proliferative characteristics, they likely still pose a lower oncogenic risk than stem cells. On the other hand, porcine pancreatic islets raise concerns regarding the risk of infectious disease transmission. Porcine islets carry the risk of transmitting porcine endogenous retroviruses (PERVs) to humans. However, extensive research and clinical trials have shown no evidence of PERV transmission so far, which is encouraging for their safety profile [121–125]. Nonetheless, the long-term risks of zoonotic disease transmission remain a concern that requires ongoing surveillance [126]. One of the key challenges limiting the widespread adoption of human islet allo-transplantation in countries like the USA is the stringent regulatory framework [127]. While porcine islets are subject to established xenotransplantation guidelines, which include rigorous safety and ethical evaluations, it is not entirely clear if they are better positioned to meet these requirements compared to human islets. Genetic modifications to reduce xenoreactivity and address safety concerns are promising but their effectiveness in fully satisfying regulatory criteria remains to be confirmed. Additionally, while regulatory frameworks for xenotransplantation aim to facilitate approval, the comparative ease with which porcine islets might navigate these requirements versus human islets is still uncertain. Ongoing research and regulatory developments will be essential to determining whether porcine islets can more effectively meet these stringent standards. Lastly, the employment of porcine islets in certain contexts could be perceived as more ethically permissible than the use of islets derived from stem cells, contingent upon the origins of these stem cells. Specifically, the ethical controversies surrounding the utilization of embryonic stem cells are not relevant to porcine islets. Nonetheless, employing animal organs and tissues introduces distinct ethical challenges, such as those pertaining to the welfare of animals involved in breeding and procurement processes, as well as cultural and religious considerations. Regulatory agencies have established frameworks for xenotransplantation [128], which includes the transplantation of porcine islets. These frameworks address both safety and ethical issues, but they also mean that porcine islets must undergo rigorous preclinical and clinical testing to prove their safety and efficacy. In comparison, human islets and stem cell-derived islets, while also subject to strict regulatory scrutiny, are not confronted with the same level of concern regarding zoonotic disease transmission.

## Conclusion

The quest for a cure for T1D through beta cell replacement therapy encompasses a dynamic interplay of potential and complexities. The efficacy of islet transplantation from human donors underscores the foundational promise of this approach by directly addressing T1D’s root cause. However, the realization of a universal cure is hindered by several critical challenges: the quest for an inexhaustible and high-quality source of beta cells, the minimization or elimination of reliance on immunosuppressive drugs, and the assurance of the long-term viability and functionality of the transplanted cells. Alternative sources such as porcine islets and stem cell-derived islets offer intriguing possibilities, each with its unique set of advantages and challenges. Porcine islets, while providing a potentially unlimited supply, raise concerns regarding physiological compatibility, immunogenicity, and regulatory complexities. On the other hand, stem cell-derived islets, benefiting from advancements in cellular reprogramming and differentiation, appear to have a slight edge in current discussions, primarily due to their potential for unlimited supply and reduced ethical concerns compared to embryonic stem cells. However, the recent successes in whole organ xenotransplantation inject a renewed vigor into the exploration of porcine islets [129–135]. These advancements may pave the way for addressing some of the longstanding issues in a relatively short timeframe, particularly those related to immunogenicity and physiological compatibility [136]. This progress, alongside the ongoing refinement of techniques for stem cell-derived islets, underscores a dynamic research landscape. Thus, the future of beta cell replacement therapy for T1D remains an open field of scientific inquiry and innovation. It is propelled by the collective goal of developing a comprehensive, effective cure, navigating through the interwoven challenges of supply, compatibility, safety, and regulatory acceptance. The path forward is marked by the potential of recent breakthroughs and the promise of overcoming current limitations through concerted research efforts.
